# Intrinsic Properties of Larval Zebrafish Neurons in Ethanol

**DOI:** 10.1371/journal.pone.0063318

**Published:** 2013-05-03

**Authors:** Hiromi Ikeda, Alison H. Delargy, Tohei Yokogawa, Jason M. Urban, Harold A. Burgess, Fumihito Ono

**Affiliations:** 1 Section on Model Synaptic Systems, Laboratory of Molecular Physiology, National Institute on Alcohol Abuse and Alcoholism, National Institutes of Health, Rockville, Maryland, United States of America; 2 Unit on Behavioral Neurogenetics, National Institute of Child Health and Development, National Institutes of Health, Bethesda, Maryland, United States of America; University Zürich, Switzerland

## Abstract

The behavioral effects of ethanol have been studied in multiple animal models including zebrafish. Locomotion of zebrafish larvae is resistant to high concentrations of ethanol in bath solution. This resistance has been attributed to a lower systemic concentration of ethanol in zebrafish when compared with bath solution, although the mechanism to maintain such a steep gradient is unclear. Here we examined whether the intrinsic properties of neurons play roles in this resistance. In order to minimize the contribution of metabolism and diffusional barriers, larvae were hemisected and the anterior half immersed in a range of ethanol concentrations thereby ensuring the free access of bath ethanol to the brain. The response to vibrational stimuli of three types of reticulospinal neurons: Mauthner neurons, vestibulospinal neurons, and MiD3 neurons were examined using an intracellular calcium indicator. The intracellular [Ca^2+^] response in MiD3 neurons decreased in 100 mM ethanol, while Mauthner neurons and vestibulospinal neurons required >300 mM ethanol to elicit similar effects. The ethanol effect in Mauthner neurons was reversible following removal of ethanol. Interestingly, activities of MiD3 neurons displayed spontaneous recovery in 300 mM ethanol, suggestive of acute tolerance. Finally, we examined with mechanical vibration the startle response of free-swimming larvae in 300 mM ethanol. Ethanol treatment abolished long latency startle responses, suggesting a functional change in neural processing. These data support the hypothesis that individual neurons in larval zebrafish brains have distinct patterns of response to ethanol dictated by specific molecular targets.

## Introduction

Alcoholism is a severe disease affecting approximately 18 million people in the United States [1]. Alcohol induces a variety of effects on the central nervous system, and acute administration of ethanol in humans induces euphoria, sedation and hypothermia [2]. With chronic ethanol exposure, neuronal adaptation causes physical dependence and tolerance as well as neurotoxicity. Various animal models have been utilized to clarify the mechanisms of ethanol induced-changes in the CNS. Recently, zebrafish (*Danio rerio*) emerged as a model in this field [3]. Zebrafish genes are 70–80% identical to human orthologs [4]. In addition, their CNS possesses classical vertebrate architecture and a full complement of neurotransmitters [5], [6]. Thanks to these similarities, zebrafish are being increasingly utilized as a model in behavioral pharmacology.

Zebrafish exhibit a variety of behavioral changes induced by ethanol exposure including social behavior (shoaling and aggression), light/dark preference, and locomotor activity [3], [7]–[10]. One striking feature of zebrafish larvae is the resistance of their locomotion to very high doses of ethanol. While the mechanism remains unclear, the resistance has been attributed to robust metabolism of ethanol resulting in a lower systemic ethanol concentration. In all previous studies, ethanol was administered by soaking the whole larvae in solutions containing ethanol. However, it is difficult to determine whether the systemic concentration of ethanol in larvae approximates the concentration in the bath solution. Several biochemical studies examined the concentration of ethanol in homogenized larvae after ethanol exposure but the results were variable depending on the preparation [7], [8], [11].

In this study, we examined the intrinsic properties of larval zebrafish neurons using a new preparation designed to better control the internal ethanol concentration of zebrafish by minimizing contributions of metabolism and diffusional barriers. Under these conditions, zebrafish neurons displayed a variety of responses to ethanol and some neurons showed resistance to exceptionally high concentrations of ethanol.

## Materials and Methods

Fish maintenance and breeding. Zebrafish (*Danio rerio*) larvae used for the behavioral analysis were of the TLF (Tubingen long fin) strain. For calcium imaging, siblings from crossings of male and female adults (TLF or AB) were divided into control and treatment groups. Adult fish were maintained in stand-alone, self-circulating systems (Aquatic Ecosystems and TECNIPLAST) in the animal facility at the National Institute on Alcohol Abuse and Alcoholism (NIAAA) following National Institutes of Health (NIH) Animal Care and Use Committee guidelines (Permit number: LMP-FO-11). Embryos were collected in the morning and thereafter maintained at 28°C. Experiments were conducted at 6 days post-fertilization (dpf), unless otherwise indicated. All larvae were used for experiments at stages before their sex was determined.

Behavior recording and kinematic analysis. Video recording and locomotion kinematic analysis was performed as described previously [12], [13]. Briefly, images at 512×512 resolution were collected with a Photron high speed camera at 1000 frames/s. Experiments were carried out at 25–28°C with the experimental setup isolated by a black shroud. Larvae were illuminated using a custom built array of infrared (880 nm peak) LEDs (Stackley Devices). Larvae were studied in groups of 20–30 in 6 cm Petri dishes mounted on a mini-shaker (Bruel and Kjaer). Vibration of the mini-shaker was controlled by computer-generated waveforms for startle response experiments [13]. Startle responses (C-starts) were identified by changes in body orientation >16°C over a 3 ms window [13]. Individual responses were shown as latency histograms. Alternatively the percentage of larvae displaying the startle response was calculated for each Petri dish. Automated tracking software written in the Interactive Data Language (IDL Visual Information Systems) was used for locomotion detection and analysis.

Calcium imaging of reticulospinal neurons. Mauthner, vestibulospinal and MiD3 neurons were labeled by backfilling, following previous studies with modifications [14]–[16]. Briefly, a fluorescent Ca^2+^ indicator, Calcium Green dextran (10,000 molecular weight; Invitrogen) was injected into the spinal cord of 5 dpf larvae. After the injection, larvae were allowed to recover in bath solution (NaCl 110 mM, HEPES 5 mM, CaCl_2_ 2 mM, glucose 3 mM, KCl 2 mM and MgCl_2_ 0.5 mM, pH 7.4) for >15 h. Larvae were mounted in an upright position with the dorsal side facing the objective. This was accomplished using low-melting point agarose (2.5%; Nacalai Tesque) in glass-bottom 35 mm plastic dishes. For the hemisected preparation, mounted larvae were cut at the level of the 10^th^ body segment and the caudal portion removed. Intact sibling larvae were used as controls. The dish was fixed with dental wax to a hole drilled in a plastic plate (165×260×2 mm) to which an audio speaker (SC5.9, Art.No. 8006; VISATON) was attached with screws. A silicon gel sheet (V30Z62MCH100230; Advanced Antivibration Components) supported the four corners of the plate. Imaging was performed on a LSM510 Meta confocal microscope (Carl Zeiss Microscopy) with a 40×1.2 NA water-immersion objective at 256×256 resolution. Mauthner, vestibulospinal, and MiD3 neurons were illuminated with a 488 nm argon laser line and sequential confocal images (at 250 ms intervals) were acquired for ROIs encircling the neurons. Vestibulospinal neurons constitute a compact group and the ROI was chosen so that it encompasses all labeled neurons. For MiD3 neurons, the ROI encompassed MiD3cm and MiD3i neurons [17]. The pinhole was fully open which provided an optical slice of 11.8 μm. A TTL signal generated by the Zeiss Zen software was used to trigger the sound/vibration stimulation. A stimulation waveform (150 ms, 500 Hz) generated by a custom made oscillator (Stackley Devices) was routed to an audio amplifier prior to driving a speaker. To ensure that the increase in fluorescence intensity was not caused by a focal plane shift, we chose the focal plane with the highest intensity before each trial. In experiments with multiple cells in the field, we picked a focal plane where the cells of interest were at or near their maximum intensity. All quantification was performed in Zeiss Zen software. Fluorescence intensities of cell bodies were measured and the relative changes in fluorescence from the resting intensity (ΔF/F) were calculated and plotted. The frame during the vibration was excluded from the analysis due to mechanical artifacts, as in other zebrafish studies of calcium imaging using mechanical stimuli [14], [16], [18]. To image the cranial vasculature, larvae in the anterior segment preparation or intact larvae were incubated in bath solution containing 0.6 mg/ml Alexa 488 dextran (10,000 molecular weight; Invitrogen) for 30 min before confocal microscopy.

Histology. Larvae were anesthetized with MS-222 and fixed with 4% PFA at 4°C for 12–16 hrs. After washing with PBS containing 20% sucrose at 4°C overnight, the fixed larvae were embedded in a 1∶1 mixture of Tissue Tek OCT compound (Sakura Finetech) and 20% sucrose. Samples were then frozen and sectioned (16 μm) on a cryostat. Sections were mounted on Superfrost Plus slides (VWR) and dried. After rinsing in tap water, the Nova Ultra H&E Stain Kit (IHC world) was used for hematoxylin and eosin stain.

Acridine orange (AO) staining. Apoptotic cells in larvae were detected using AO [19]. After treatment with 0 or 300 mM ethanol for 1 h, larvae were washed twice in bath solution, and transferred to bath solution containing 5 μg/ml AO. After staining for 1 h at room temperature followed by eight 5-minute washes in bath solution, the larvae were anesthetized with 0.01% MS-222, mounted in 2% low-melting point agarose and examined by confocal microscopy.

Statistical analysis. Statistical analyses were performed using Excel (Microsoft) and Prism 5 (GraphPad Software). After analysis of variance (ANOVA), significant results followed by Bonferroni-corrected t tests between groups are indicated in the text. Graphs show average and standard error of the mean.

## Results

### Zebrafish larvae in a high concentration of ethanol exhibit hyperactivity

**Figure 1:**
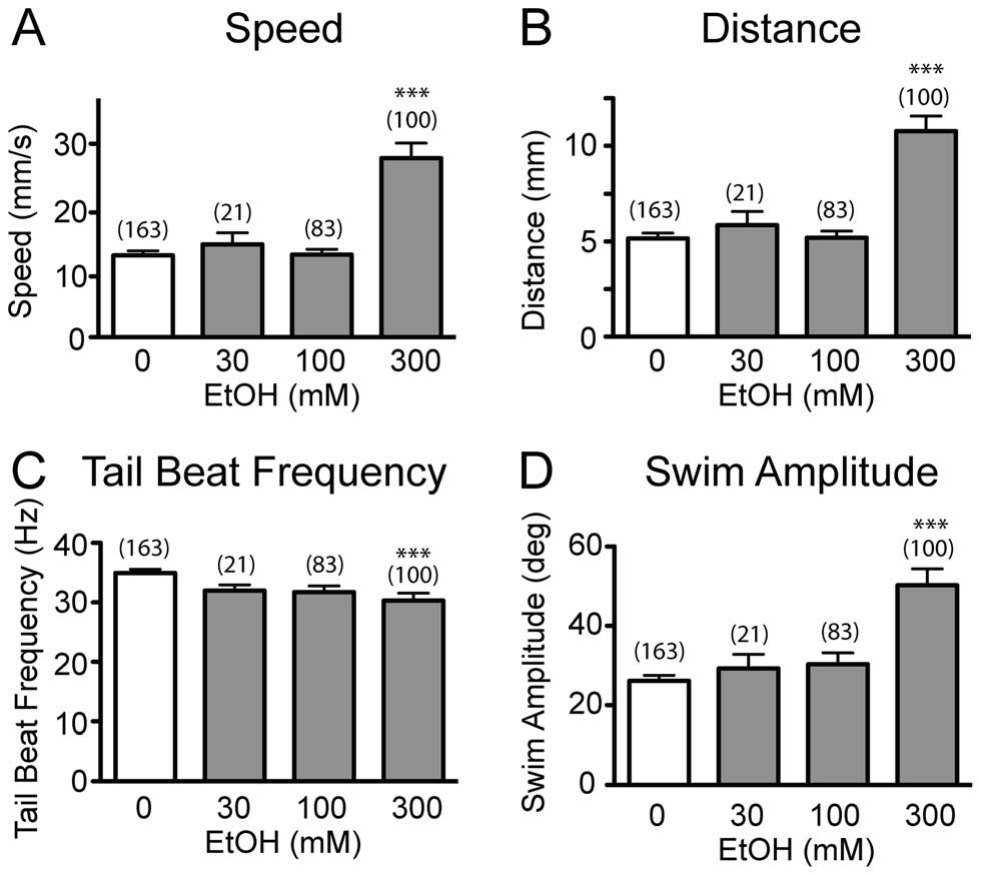
The effects of ethanol on spontaneous locomotion. Spontaneous locomotion kinematics of larval zebrafish (n = 20–25) after exposure to 0, 30, 100 and 300 mM ethanol for 30 min. Speed, distance, tail beat frequency, and swim amplitude are shown in A, B, C, and D, respectively. Numbers of analyzed larvae are shown in brackets (***P<0.001 versus 0 mM ethanol).

We initially investigated the movement of free-swimming larvae in response to ethanol. The kinematics of spontaneous locomotion of 6 day post-fertilization (dpf) larval zebrafish were examined using automated motion detection and analysis software after 30 min incubation in 0–1000 mM ethanol [8], [13], [20]. In 1000 mM ethanol, larvae exhibited sedation after brief hyperactivity and died within 3 h. Larvae in 0–300 mM were subjected to kinematic analysis. The effect of ethanol on swim kinematics was robust and dose-dependent. While the tail beat frequency decreased slightly in response to 300 mM ethanol (Fig. 1C), the amplitude of tail beats displayed a robust increase (Fig. 1D), resulting in much greater swim speed (Fig. 1A) and distance traveled per swim bout (Fig. 1B). In humans, 10 mM (0.05%w/v) Blood Alcohol Concentration (BAC) produces stimulant effects (hostility-aggression and happiness) while BAC >20 mM (0.10%w/v) has depressant effects. BAC >68 mM (0.4%w/v) is often lethal. In contrast, the hyperactivity exhibited by zebrafish larvae bathed in 300 mM ethanol indicates their resistance, which largely agrees with previous studies.

### Controlling the internal ethanol concentration by allowing the free access of bath solution

We hypothesized that the properties of neurons, in addition to the metabolic ability of zebrafish larvae, may play roles in ethanol resistance. In order to test this hypothesis, we attempted to better control the internal ethanol concentration of larvae by sectioning the larval zebrafish into cranial and caudal halves. The cranial half was soaked in ethanol solution. We reasoned that chemicals in the bath solution might gain free access to the blood vessels exposed in the section plane thereby fixing the internal concentration to that of the bath solution.

**Figure 2:**
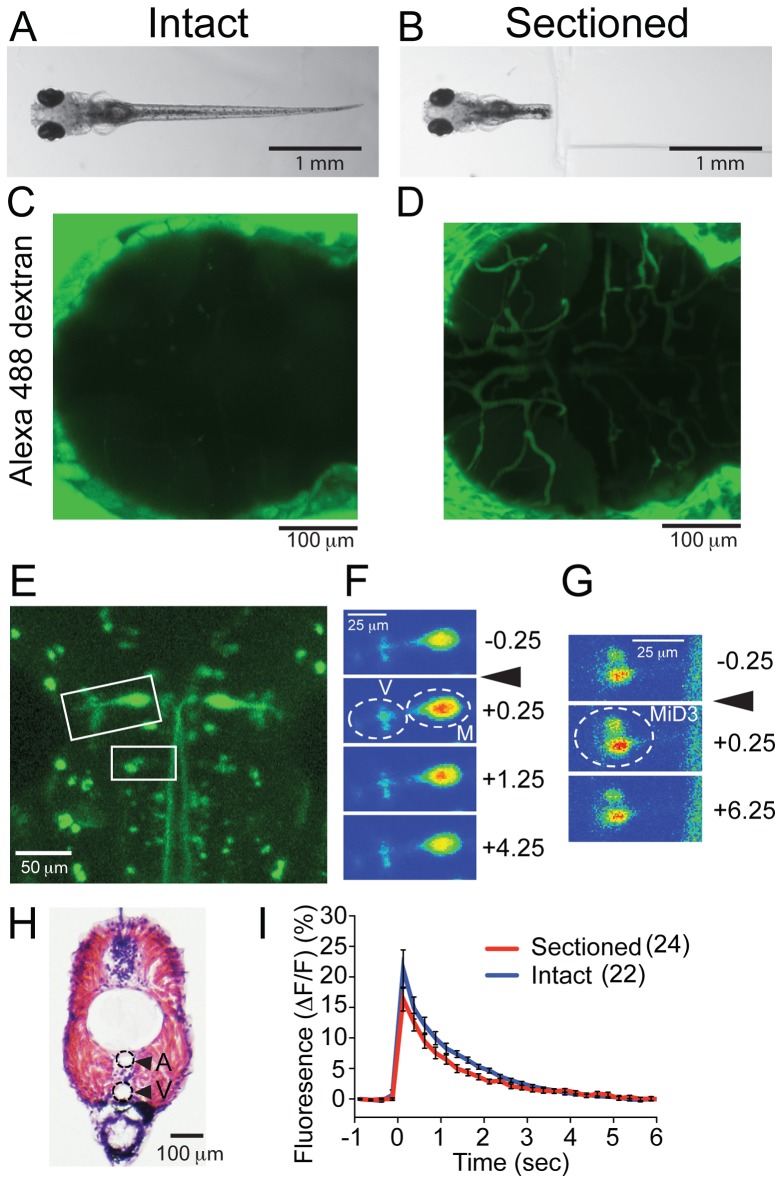
Sectioned larvae compared to intact larvae (A, B). Intact (A) and sectioned larva (B) were immersed in Alexa 488 dextran solution for 30 min. (C, D) Fluorescence images obtained from the cranial region after incubation. In the sectioned preparation, fluorescent dye stained the vasculature. (E–G) Calcium imaging of reticulospinal neurons. Reticulospinal neurons labeled with dextran-conjugated calcium green (E). Intensities of calcium green signal in boxed areas are shown in F and G as pseudocolors. Mauthner neurons (M), vestibulospinal neurons (V) and MiD3 neurons (MiD3) displayed increase of [Ca^2+^]_i_ after the sound/vibration stimuli (arrowheads). Signals were measured in areas encircled by dashed lines before (−0.25 sec) and after the stimuli (+0.25, +1.25, +4.25, +6.25 sec). (H) Image of HE stained larva trunk at the 9^th^ body segment level. A and V designate artery and vein, respectively. (I) Plots of the fluorescence intensity change in the Mauthner neurons with sound/vibration stimuli. Traces from intact (blue) and sectioned larvae (red) are shown. Six Mauthner cells were measured in each condition. Stimuli were applied and calcium transients were measured at each concentration 2–5 times. The numbers of samplings are shown in parentheses.

We first examined the access of chemicals in the bath solution to cerebral blood vessels by using a fluorescent molecule. When 6 dpf intact larvae were bathed in solution containing Alexa 488 dextran (10,000 MW), no fluorescence was observed inside the crania after 30 min incubation (Fig. 2A, C). On the other hand, when the cranial half of a larva was embedded in agarose with the section plane exposed to the bath solution (Fig. 2B) for 30 min, anatomical structures resembling cephalic blood vessels were clearly visualized with fluorescence. The labeled structures in Fig. 2D are consistent with blood vessels seen previously in the detailed anatomy of vasculature of a 4.5 dpf larva [21]. The dorsal aorta and the posterior cardinal vein (diameters are >20 μm) are visible in the plane at the 9^th^ body segment (Fig. 2H). These data suggest that small water-soluble molecules such as ethanol in the bath solution will gain access to cephalic blood vessels through the severed end of the major vessels.

Before examining the preparation in ethanol, we tested whether neuronal function was also maintained in isolated cranial sections. Calcium green dextran was injected into the spinal cord of 5 dpf larvae. After an overnight recovery in the bath solution, reticulospinal neurons were labeled with calcium green (Fig. 2E). We measured the changes in cytosolic free Ca^2+^ concentration ([Ca^2+^]_i_) from Mauthner neurons. The cranial halves of larvae were mounted on a glass-bottom dish, placed in the sound/vibration apparatus, and covered with ethanol-free bath solution. When the vibration reached the larva, sensory cells were stimulated and the signal was transmitted to reticulospinal neurons [14]–[16]. Within 250 ms following the trigger, a robust increase of calcium green fluorescence intensity was observed in labeled Mauthner neurons. The signal gradually returned to the basal level (Fig. 2F). Calcium signals were also observed in other types of reticulospinal neurons, including vestibulospinal neurons and MiD3 neurons (Fig. 2F, G). Responses of Mauthner neurons were compared to intact larvae. Remarkably, the [Ca^2+^]_i_ responses were very similar between intact and sectioned larvae (Fig. 2I). These findings show that the functional integrity of the neural network is retained after sectioning.

### Sensitivity of reticulospinal neurons to ethanol

**Figure 3:**
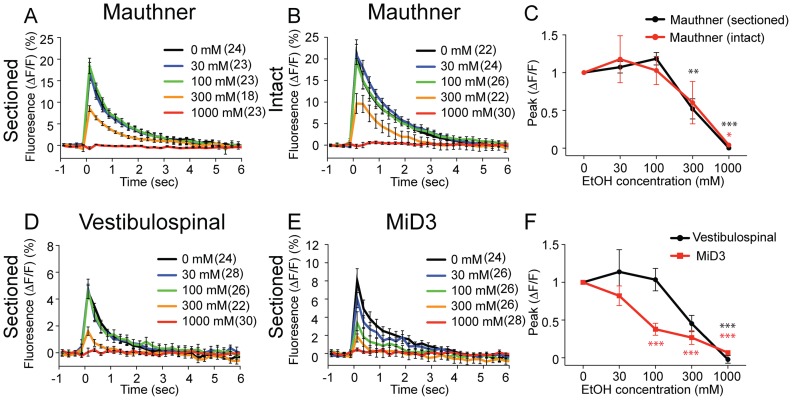
Activity of reticulospinal neurons in ethanol. Fluorescence intensity of Mauthner neurons (A–C), vestibulospinal neurons (D, F) and MiD3 neurons (E, F). Sound/vibration stimuli were applied to sectioned (A, D, E) or intact larvae (B) bathed in 0, 30, 100, 300 and 1000 mM ethanol. The black, blue, green, orange and red lines correspond to 0, 30, 100, 300, and 1000 mM ethanol, respectively. The peak of ΔF/F normalized to values at 0 mM was plotted against the EtOH concentration in C and F. (*P<0.05, **P<0.005, ***P<0.001 versus 0 mM ethanol). Six Mauthner neurons, eight vestibulospinal neurons, and five MiD3 neurons were measured. Stimuli were applied and calcium transients were elicited at each concentration 2–5 times. The numbers of samplings are shown in parentheses.

We bathed the sectioned cranial half of larvae in bath solution containing varying concentrations of ethanol (0, 30, 100, 300 and 1000 mM) and measured activities in 3 types of reticulospinal neurons, i.e. Mauthner neurons, vestibulospinal neurons and MiD3 neurons. Mauthner neuron [Ca^2+^]_i_ transients were not significantly altered in 30 and 100 mM ethanol, decreased in 300 mM ethanol, and were eliminated in 1 M ethanol (Fig. 3A, C). In vestibulospinal neurons, the overall effect was similar to that of Mauthner neurons (Fig. 3D, F). MiD3 neurons were more sensitive to ethanol compared to the other two neuronal types, showing a marked decrease at 100 mM ethanol resulting in a leftward shift of the dose response curve (Fig. 3E, F).

We also compared the [Ca^2+^]_i_ responses of the Mauthner neuron in sectioned preparation to those measured in intact larvae. Unexpectedly, the dose response curves in the two preparations were almost identical (Fig. 3A–C). This suggests that the ethanol concentration surrounding neurons in the intact larvae approximates that of the bath solution. Consequently, the intrinsic properties of neurons likely contribute to the decreased potency of ethanol.

### Recovery and adaptation of neural activity in ethanol

**Figure 4:**
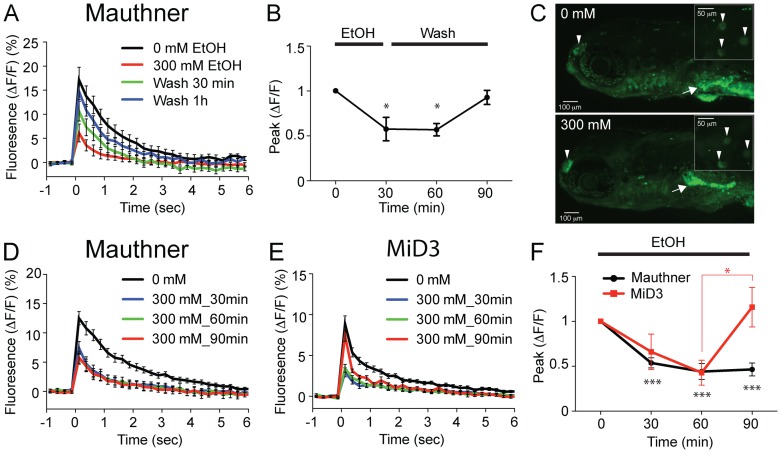
Recovery and adaptation of neuronal activity in 300 **mM ethanol.** Fluorescence intensities of Mauthner neurons and MiD3 neurons were measured in response to sound/vibration stimuli. Sectioned larvae were either washed after 30 min incubation of 300 mM ethanol (A) or bathed continuously in 300 mM ethanol (D, E). Black, red, green and blue traces correspond to 0, 30, 60 and 90 min, respectively. The peaks of ΔF/F normalized to 0 min were plotted against time in B (for A) and F (for D, E) (*P<0.05, ***P<0.001 versus 0 min). Five Mauthner neurons and six MiD3 neurons were measured. Stimuli were applied and calcium transients were elicited at each concentration 2–5 times. The numbers of samplings are shown in parentheses. (C) AO staining in 0 mM (top) and 300 mM (bottom) ethanol are shown. Apoptosis in olfactory organs is marked with arrowheads. Apoptosis of neuromasts, located on the body surface, are shown with arrowheads in insets. Nonspecific signals are observed in the gut (Arrows).

We examined whether the effects of 300 mM ethanol on zebrafish neurons were reversible. When the sectioned preparation was bathed continuously in 300 mM ethanol, the ΔF/F peak in Mauthner neurons showed a marked decrease between 0 and 30 min, and remained stable after 30 min (Fig. 4D, F). In contrast, when washed after 30 min, Mauthner neurons displayed a robust recovery approaching the level of the pre-ethanol treatment (Fig. 4A, B). This suggests that 300 mM ethanol does not cause an irreversible effect on Mauthner neurons. To further examine the effect of 300 mM on neurons other than Mauthner neurons, AO staining was performed in 4 dpf larval zebrafish after 1 hour exposure of 300 mM ethanol. AO binds to DNA in apoptotic cells and is used widely as a marker of apoptosis in zebrafish [22], [23]. The olfactory organ and neuromasts showed apoptosis in untreated as well as ethanol treated larvae, as was previously reported using TUNEL assays [24]. Unlike chronic exposure of ethanol [19], increase of apoptosis was not observed after 1 hr exposure to 300 mM ethanol, even in larvae with a relatively high level of olfactory organ apoptosis (Fig. 4C).

Interestingly, the time course of MiD3 neuron activity in 300 mM ethanol displayed quite a different pattern. The response, after decreasing at 30 and 60 min, showed a robust recovery at 90 min without wash (Fig. 4E, F) despite the continued presence of ethanol. Because the measurement was performed in the sectioned preparation with a controlled ethanol concentration, the recovery of neural activity likely arose from the adaptation of neurons to 300 mM ethanol.

### Change of startle response in ethanol

**Figure 5:**
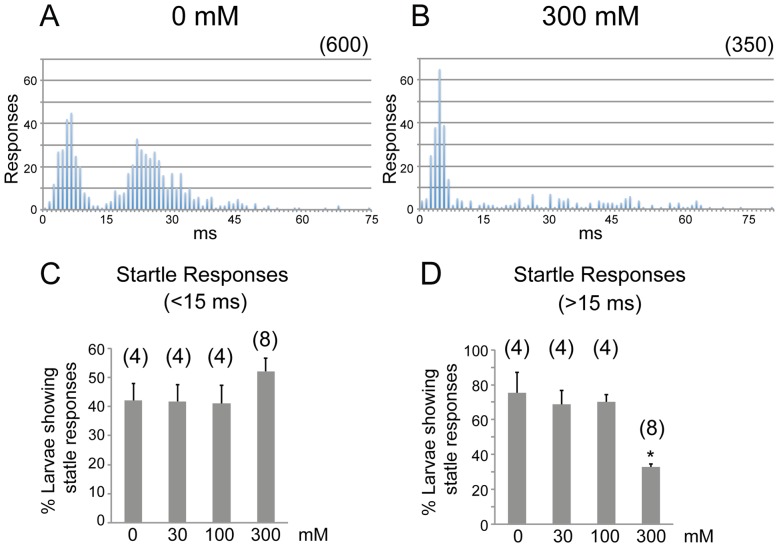
The effects of ethanol on acoustic startle responses. Histograms showing the latency distribution of startle responses of free-swimming larvae after 1 hr exposure to 0 (A) and 300 mM ethanol (B). N in parenthesis is the number of responses analyzed (from a total of 100–120 larvae each). Percentage of larvae showing responses with latencies <15 ms (C) and >15 ms (D) after exposure to 0, 30, 100 and 300 mM ethanol. (* P<0.001 t-test compared to 0 mM group). N = 4 groups of 20 larvae (0, 30, 100 mM treatments) or 8 groups of 20 larvae (300 mM treatment).

Reticulospinal neurons including Mauthner neurons and MiD3 neurons are activated in startle responses [14]. We therefore examined whether the ethanol-induced changes in reticulospinal neuron activity affect acoustic startle responsiveness. After 1 hr treatment in 0, 30, 100 and 300 mM ethanol, larvae were tested with mechanical vibration while monitoring startle responses. Reponses were identified based on kinematic parameters matching C-starts [13]. As previously described, two types of startle responses were observed in untreated larvae, distinguished by latency, one centering around 7–8 ms (short latency C-start, ‘SLC’) and the other centering around 28 ms (long latency C-start, ‘LLC’) (Fig. 5A) [13]. 300 mM ethanol treatment did not affect SLC responsiveness (F[3], [16] = 1.4, p = 0.29), but selectively reduced LLC responsiveness (F[_3]_, [16] = 26.6, p<0.001). While the 30 mM and 100 mM ethanol treatments did not produce differences (Fig. 5C, D), in 300 mM treated larvae LLC responses were greatly diminished (Fig. 5B, D). Differential sensitivity of reticulospinal neurons to ethanol likely contributes to the behavioral shift seen in 300 mM ethanol.

## Discussion

In this study we examined the intrinsic properties of neurons in zebrafish larvae exposed to high doses of ethanol in a semi-intact preparation. The absorption and degradation process of ethanol in zebrafish larvae remains largely unexplored, and the systemic concentration of ethanol in zebrafish relative to bath solution has been controversial. Comparison of the neural activity in sectioned and intact larvae suggest that the ratio of internal/external ethanol concentration in 5–6 dpf zebrafish larvae may be close to 1. This is consistent with the solubility of ethanol in both aqueous and lipid environments and the ease with which ethanol crosses biological membranes.

We used Mauthner, vestibulospinal, and MiD3 neurons to investigate effects of ethanol because these neurons displayed consistent calcium signals in response to stimuli. Mauthner and MiD3 neurons are involved in the startle responses [25] while the functional role of vestibulospinal neurons is less clear. Although it has not been determined which reticulospinal neurons are involved in spontaneous swimming, the inability of high ethanol concentrations to impair this behavior (Fig. 1) suggests that involved neurons are also resistant to >100 mM ethanol. Startle responses after exposure to 300 mM ethanol exhibited an unexpected change. In normal conditions, SLC responses are controlled by Mauthner neurons, while the cellular underpinnings of LLC responses remains unclear [13]. The current data suggests that neurons responsible for LLC responses may be more sensitive to ethanol than Mauthner neurons. However, it requires further investigation whether the single peak in 300 mM ethanol treated larvae represents bona fide SLC responses. First, the mean latency of the group is slightly faster than for SLC responses in untreated larvae (t-test p<0.001, Fig. 5A, B). Second, the activity of Mauthner neurons in 300 mM as indicated by Calcium Green was reduced, though it may still be sufficient to generate action potentials. It is therefore possible that the neuronal basis of SLC responses is different in larvae treated with 300 mM ethanol. The variety of resistance and adaptation observed among reticulospinal neurons in larval zebrafish was unexpected. Further investigations of proteins that are differentially distributed and responsible for these differences, e.g. between Mauthner and MiD3 neurons, may provide insight into the neurobiology of ethanol effects. While chronic exposure to 30–100 mM of ethanol throughout the development leads to apoptosis of neurons and genetic/behavioral changes [19], [26], the resistance of some zebrafish neurons to acute exposure of >100 mM ethanol is unusual, when compared to mammalian neurons. Therefore, comparison of zebrafish to other model systems at the level of genetics and physiology may allow identifications of discrete molecules underlying the resistance and adaptation of zebrafish neurons to ethanol. The easily measurable and stereotyped locomotion of larval zebrafish, when combined with genetic, optical, and electrophysiological techniques readily applicable in zebrafish, should facilitate identification of factors determining the sensitivity and the adaptation of neurons to ethanol.
